# Infection-embedded penicillin allergy de-labelling delivers immediate antimicrobial stewardship gains in patients with active infection

**DOI:** 10.1093/jacamr/dlag189

**Published:** 2026-09-04

**Authors:** Stephanie Harris, Akish Luintel, Sarah Jones, Gabriella Hardy-Gould, Neil Reynolds, Preet Panesar, Melina Makatsori, Joanna Lukawska, Indran Balakrishnan, Alex Kew, Cassandra Watson, Alastair McGregor, Vanya Gant, Trupti Patel, Abhishek Das

**Affiliations:** Department of Clinical Microbiology, University College London Hospitals, London, UK; Department of Clinical Microbiology, University College London Hospitals, London, UK; Department of Pharmacy, University College London Hospitals, London, UK; Department of Pharmacy, University College London Hospitals, London, UK; Department of Pharmacy, University College London Hospitals, London, UK; Department of Pharmacy, University College London Hospitals, London, UK; Department of Specialist Allergy and Clinical Immunology, University College London Hospitals, London, UK; Department of Specialist Allergy and Clinical Immunology, University College London Hospitals, London, UK; Department of Microbiology, Royal Free London NHS Foundation Trust, London, UK; Department of Microbiology, Royal Free London NHS Foundation Trust, London, UK; Hospital for Tropical Diseases, University College London Hospital, London, UK; Department of Pharmacy, London North-West University Healthcare NHS Trust, London, UK; Department of Infectious Diseases, London North-West University Healthcare NHS Trust, London, UK; Department of Clinical Microbiology, University College London Hospitals, London, UK; Department of Clinical Microbiology, University College London Hospitals, London, UK; Department of Clinical Microbiology, University College London Hospitals, London, UK; Hospital for Tropical Diseases, University College London Hospital, London, UK; Division of Infection and Immunity, University College London, London, UK

## Abstract

**Objectives:**

Unconfirmed penicillin allergy labels restrict antibiotic options and drive deviation from antimicrobial guidelines. To address this, we established an infection-embedded penicillin allergy de-labelling (PADL) service embedded within existing infection-liaison touchpoints (microbiology meetings, advice calls and infection reviews) to target patients with active infection needs.

**Methods:**

We performed a retrospective analysis of all 189 referrals to our PADL service (March 2022 to December 2025), nested within a baseline prevalence review of 218 375 admissions. Furthermore, to quantify the clinical cost of allergy labels, we surveyed infection experts, using 20 clinical vignettes, to determine prescribing variance had de-labelling not occurred.

**Results:**

Of patients, 82.5% (156/189) were classified as having acute, complex or recurrent infection; 45% (85/189) were directly de-labelled, while 41.8% (79/189) were excluded by low-risk criteria prompting specialist referral for full penicillin allergy testing. Critically, for patients on active therapy, de-labelling triggered an immediate regimen change in 84.1%, driving a 43% intravenous-to-oral switch rate, a mean reduction of 0.7 antibiotics per patient and significantly reducing antibiotic spectrum coverage (*P* = 0.0004). Expert review of vignettes revealed only minimal concordance on alternative regimens (Fleiss’ kappa 0.28) had we not de-labelled, implying that persistent labels lead to non-standardized prescribing.

**Conclusions:**

Infection-embedded de-labelling is a high-yield intervention that can deliver immediate stewardship returns in patients with active infection needs. A caveat is that a significant proportion of excluded patients require specialist allergy referral, carrying resource implications and underscoring the need for careful planning with local allergy services at inception of ‘low-risk’ pathways.

## Introduction

Penicillin allergy is reported in up to 15% of UK hospital inpatients,^1–5^ yet targeted history-taking and appropriate testing show that >95% of labels can be safely removed.^6–8^ Many arise from non-allergic events including drug intolerance (e.g. gastrointestinal symptoms) or non-pruritic childhood rashes that could be attributable to viral infections.^9^ Nonetheless, the assignment of a penicillin allergy ‘label’ itself can be detrimental, as it is associated with longer hospital stays,^10^ higher treatment costs,^1,11,12^ increased risk of *Clostridioides difficile* infection,^13^ increased colonization with resistant organisms and higher rates of surgical site infections.^14,15^ Indeed, penicillin allergy de-labelling initiatives have been shown to reduce use of ‘Reserve’ and ‘Watch’ category antibiotics,^16^ and facilitate more consistent alignment towards first-line antibiotic regimens and lower healthcare costs.^17^

Timely de-labelling is particularly critical for patients with complex, deep-seated or recurrent infections. These patients are routinely reviewed through existing infection-liaison structures, including microbiology multidisciplinary meetings, telephone advice and inpatient infectious diseases consultations. These touchpoints therefore provide repeated opportunities to identify allergy labelled patients precisely when antibiotic optimization is clinically urgent.

We therefore designed an infection-embedded PADL pathway that leveraged the full scope of our infection-liaison network, seeking to identify suitable low-risk candidates with active infection needs, for immediate near-patient drug provocation testing or imminent outpatient review. Our service incorporated British Society for Allergy and Clinical Immunology (BSACI) criteria^18^ and validated tools such as PEN-FAST to support risk stratification,^19^ while drawing on established models shown to be safe and effective in low-risk cohorts.^8,20–26^

This study reports the implementation and evaluation of an infection-led PADL service in 189 patients with active or ongoing infection. We frame de-labelling primarily as an antimicrobial stewardship intervention, describing a novel nested model for rapid antibiotic optimization, intravenous-to-oral switch and narrowing of antibiotic spectrum, in patients with active infection needs. Such models could complement, rather than replace, traditional ‘capture-all’ approaches.

## Methods

### Ethics

This study was a retrospective review of routinely collected clinical data as part of a service evaluation, and was not deemed to require National Health Service Research Ethics Committee approval. Patient data were used in accordance with general data protection regulations. UCL Research Ethics Committee considers anonymized online surveys without personal data and on non-sensitive topics to be exempt from ethics approval.

### Study design

Routine penicillin allergy assessment and de-labelling for low-risk cohorts were integrated into our infectious diseases service at University College London Hospital, a 1022-bed tertiary teaching hospital. The study included all adult patients (≥18 years) reviewed by our service between 11 February 2022 and 11 December 2025, targeting, but not exclusive to, patients receiving antibiotics for acute, complex or recurrent infections (definitions below) or those imminently requiring long-term penicillin prophylaxis.

Acute infection: requiring ≤14 days of antibiotics and not requiring surgical or interventional radiological management.

Complex infection: requiring >14 days of antibiotic treatment or requiring surgical or interventional radiological management.

Recurrent infection: more than two episodes of the same infection within a year, either at the same anatomical site (e.g. repeated diabetic foot infections) or with the same pathophysiology at different sites (e.g. recurrent skin and soft tissue infections due to group A *Streptococcus*).

### Referral pathway

Patients were identified through routine infection-liaison touchpoints: the daily microbiology multidisciplinary meeting, microbiology telephone consultations, inpatient infectious diseases reviews and antimicrobial pharmacist referrals. In addition, antimicrobial guidelines were updated to include a triage pathway for low-risk de-labelling, enabling ward teams to refer directly with or without infection team involvement. Referrals were received through a dedicated departmental email inbox and reviewed within one working day by a member of the PADL team, who arranged inpatient review or booked the next available outpatient appointment. Outpatient referrals were first assessed by telephone, with a face-to-face appointment for oral drug provocation testing (DPT) arranged where low-risk criteria were met.

**Figure 1.**
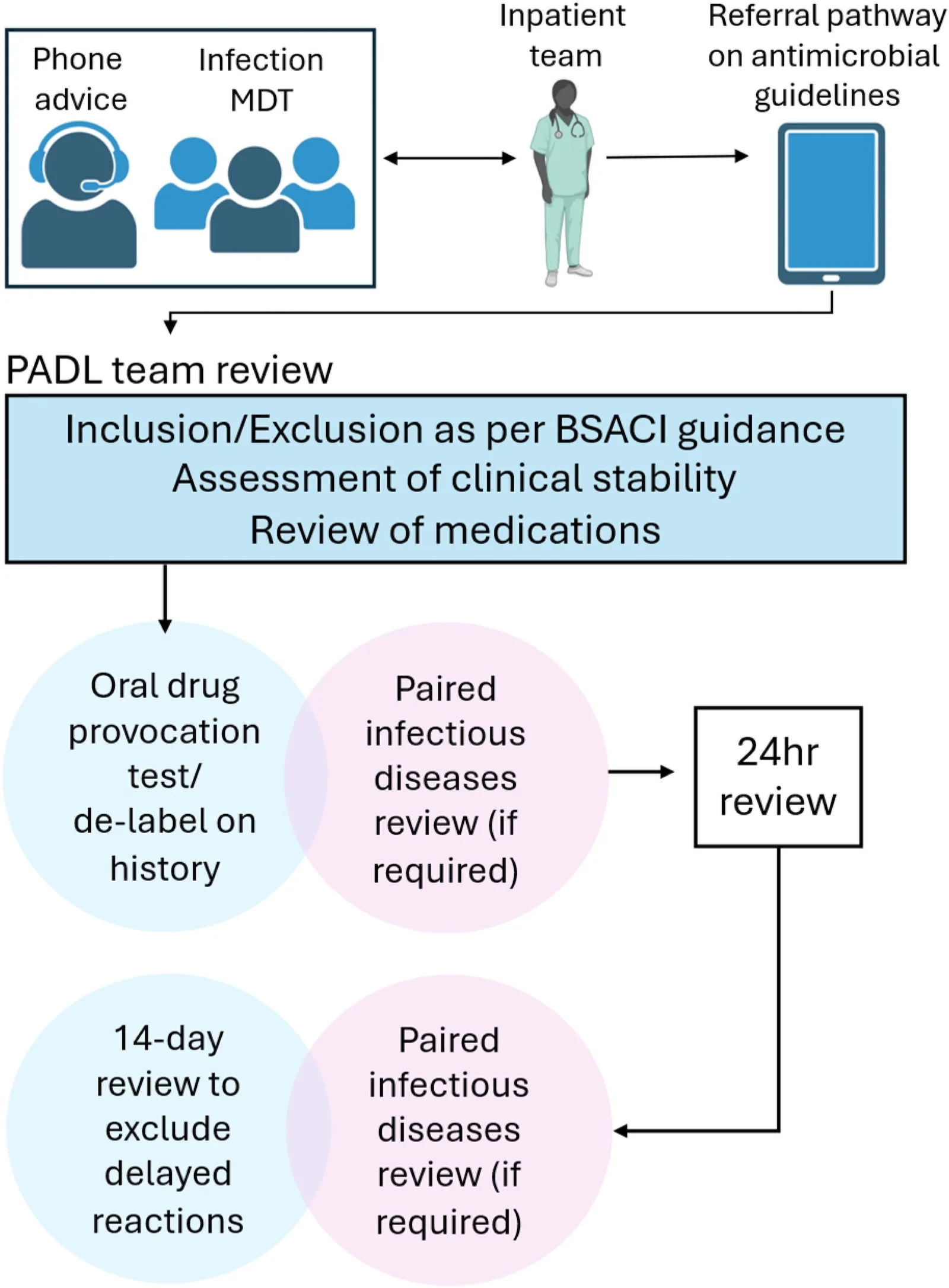
Referral pathway. Figure created with BioRender.com

The PADL team comprised two infectious diseases physicians and two senior antimicrobial pharmacists. Our trust has an established allergy-led de-labelling service to which clinicians could already refer directly, and our low-risk PADL pathway was designed to complement rather than replace this. Protocols were modelled on BSACI guidance, iterated with our Specialist Allergy and Clinical Immunology colleagues and ratified at joint infection, allergy, drug safety and antimicrobial stewardship governance meetings, which also agreed routes for onward referral and for discussion of complex cases with allergy colleagues. Team members trained by shadowing an established infection-led service, observing full allergy testing, participating in a structured induction programme and conducting five supervised challenges prior to being signed off as independent de-labellers. All referrals were reviewed and patients provided written consent before DPT. De-labelling was performed via structured history alone or oral DPT according to BSACI low-risk criteria.^18^ Where the index reaction implicated a specific penicillin, that agent was used for DPT, otherwise, amoxicillin was used in line with BSACI guidance unless a different agent (e.g. co-amoxiclav) was anticipated for ongoing therapy. Where appropriate, infection physicians concurrently optimized antimicrobial therapy unless an alternative plan had been pre-agreed or the patient was under an infectious diseases primary team. Patients received follow-up at 24 hours and 14 days through combined penicillin allergy and infection reviews (if appropriate), reducing the need for separate clinic attendances (Figure 1). The 14-day timepoint was chosen pragmatically to capture delayed reactions while allowing concurrent review of the infection episode following discharge. All patients were also given the team email address and a direct contact number to report symptoms at any point beforehand.

### Data collection

Data were extracted from electronic health records (Epic Systems Corporation, Verona, WI, USA). Baseline prevalence of penicillin allergy was determined from 218 375 admissions between 1 May 2018 and 31 August 2023, including age, sex, length of stay and reported nature of the index reaction to penicillin (Table 1). For the PADL cohort, additional variables included infection category, agent used for de-labelling, pre- and post-de-labelling antibiotic regimen, intravenous-to-oral switch and Antimicrobial Spectrum Coverage (ASC) score, a measure of antibiotic spectrum and breadth,^27^ were recorded.

**Table 1.** Penicillin allergy status spanning 218 375 hospital encounters over a ∼5-year period

	Penicillin allergic			Not penicillin allergic		
Encounters (*n* = 218 375)	25 698			192 677		
Individuals (*n* = 123 889)	13 524			110 365		
Age at admission median (IQR)	56 (37–71)			47 (33–66)		
Sex^a^	M 4445 (33%)	F 9078 (67%)	Missing 1	M 47 713 (43%)	F 62 630 (57%)	Missing 22
Length of stay, days, mean (IQR)	5.852 (0–522)			5.278 (0–779)		
Reaction recorded^b^ *n* (%)						
Unknown	7353 (56.1)			—		
Rash, itching or hives	4216 (32.1)			—		
Anaphylaxis	830 (6.3)			—		
Swelling	562 (4.3)			—		
Shortness of breath	119 (0.9)			—		
Nausea/vomiting	17 (0.1)			—		
Rash	17 (0.1)			—		
Gastrointestinal intolerance	1 (0.0)			—		

^a^For 23 patients, sex data were unavailable.

^b^Reaction recorded is the most severe reaction recorded against the penicillin allergy, only one reaction per patient is recorded.

### Case vignettes

Anonymized clinical vignettes of 20 randomly selected reviews were distributed to independent infection specialists via the British Infection Association mailing list (Supplementary material, available as Supplementary data at *JAC-AMR* Online). Reviewers were asked to recommend optimal ongoing infection management as if they were consulted either at the point of de-labelling intervention, if the patient had an active infection, or at the next infection episode (for recurrent cases). The infection specialist reviewed each case as though there was no access to a local penicillin allergy de-labelling service and they were asked to base their prescribing decisions on their clinical experience. Pre-defined antimicrobial options were provided, with free-text space for alternative suggestions.

Interrater reliability of the expert responses was assessed using Fleiss’ kappa. Kappa values were interpreted as follows: >0.91, near-perfect agreement; 0.80–0.90, strong; 0.60–0.79, moderate; 0.40–0.59, weak; 0.21–0.39, minimal and <0.20, no agreement.^28^ Any alternative management option proposed by a reviewer was considered a non-concordant response.

### Statistical analysis

Analysis was performed in Rstudio (version 2022.12.0 + 353). Continuous variables were compared using two-tailed non-parametric Mann–Whitney *U*-test for unpaired data, and Wilcoxon signed-rank test for paired data. Categorical variables were compared using *χ*^2^ tests.

## Results

### Establishing the local prevalence of penicillin allergy

We analysed 218 375 hospital encounters (123 889 individuals) and identified 13 524 patients (10.9%) with a penicillin allergy label (Table 1). Labelled patients had marginally longer hospital stays (5.85 versus 5.278 days, *P* < 0.0001) and were more often female (67% versus 57%, *P* < 0.0001). Reaction type was listed as ‘unknown’ in more than half of cases (56.1%), and anaphylaxis was recorded in 6.3%.

### Characteristics of patients referred for de-labelling

A total of 189 patients were referred to the PADL service (median age 58, interquartile range (IQR) 43–70; 55% female). At review, 33.3% had acute infection, 33.3% had complex infection and 15.9%, recurrent infection. A further 2.1% required antibiotic optimization for long-term prophylaxis e.g. for recurrent cellulitis or post-splenectomy (Table 2).

**Table 2.** Characteristics of the patients referred to the PADL service

Characteristics of referred patients (*n* = 189)	
Demographics	
Age, median (IQR), years	58 (43–70)
Female:male ratio (% female)	104:85 (55)
Infection type at review (%)	
No infection	29 (15.3)
Acute	63 (33.3)
Complex	63 (33.3)
Recurrent	30 (15.9)
Requires long-term prophylaxis	4 (2.1)

### Referral route

**Figure 2.**
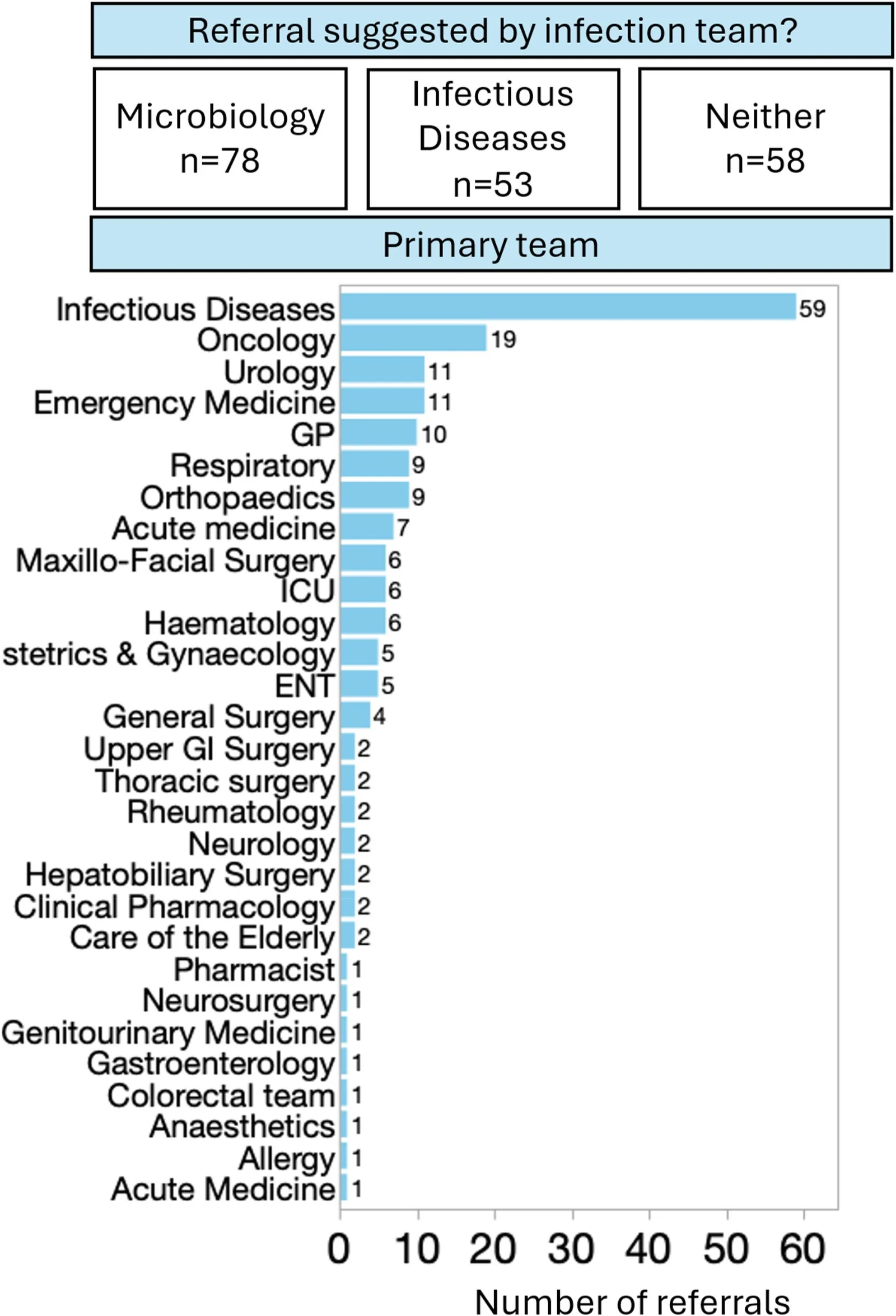
Above, number of referrals prompted by discussion with microbiology or infectious diseases team. Below, number of referrals from each team.

Here, 69% of all referrals to the PADL service were flagged by infection specialists: 41.2% (78/189) were identified by microbiology colleagues who then prompted primary teams to consult the low-risk triage pathway in our hospital antimicrobial guidelines app (Figure 2), and to refer to the PADL service via a dedicated email (Figure 1). In addition, 31% (59/189) of patients were under the infectious diseases team and referred directly, while 25% (48/189) were managed by surgical specialties (urology, orthopaedics, maxillofacial, general surgery, ear nose throat, thoracic surgery, neurosurgery and colorectal surgery) and 10% (19/189) of referrals derived from oncology teams (Figure 2). A further 30.7% (58/189) of referrals were initiated by primary teams who consulted the penicillin de-labelling section within the hospital antimicrobial guidelines app without microbiology/infection input.

### Patient outcomes

**Figure 3.**
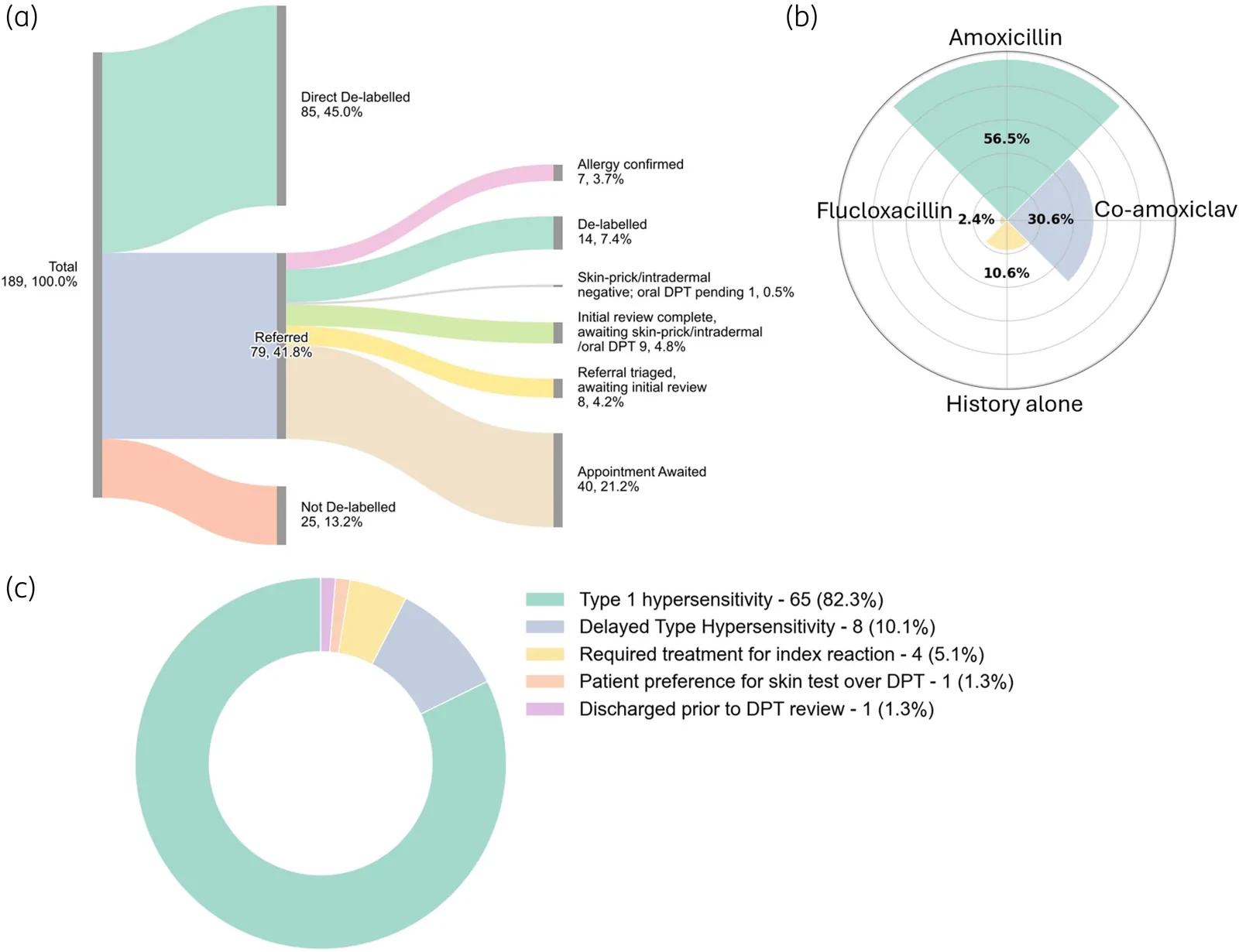
(a) Patient outcomes. (b) Agent used for de-labelling. (c) Reason for exclusion.

In total, 45% of patients (85/189) were de-labelled (Figure 3a). The most common agents for de-labelling were amoxicillin (56.5%), co-amoxiclav (30.6%) or flucloxacillin (2.4%), while 10.6% were de-labelled on history alone (Figure 3b). DPT was well tolerated (with follow-up to 2 weeks) in 84/85 cases. One patient reported isolated itching ∼45 minutes after oral DPT, without rash or other features. They were treated with oral chlorphenamine, and were asymptomatic at subsequent telephone reviews. Their allergy status was maintained and they were referred for full penicillin allergy testing (skin prick, intradermal test and supervised oral graded challenge), primarily to complete a definitive allergy work-up rather than to adjudicate the nature of the itch.

Of those patients reviewed but not de-labelled, 13.2% (*n* = 25) were excluded due to lack of consent or clinical instability at the time of de-labelling referral. The remaining 41.8% (*n* = 79) did not meet BSACI low-risk criteria and were recommended for onward full penicillin allergy testing. By December 2025, 14/79 of this group were subsequently de-labelled by allergy services, while seven had their allergy confirmed. One patient had successfully completed skin and intradermal testing, however, and was awaiting follow-on supervised oral DPT (Figure 3a).

Owing to historical features of a type I hypersensitivity reaction, 82.3% (65/79) of patients were excluded, 10.1% (8/79) due to features of a type IV reaction and 5.1% (4/79) because the index reaction had received treatment. Two patients (2.6%) were excluded due to either patient preference for full penicillin allergy testing or discharge before review (Figure 3c).

### Impact of DPT on antimicrobial prescribing

**Figure 4.**
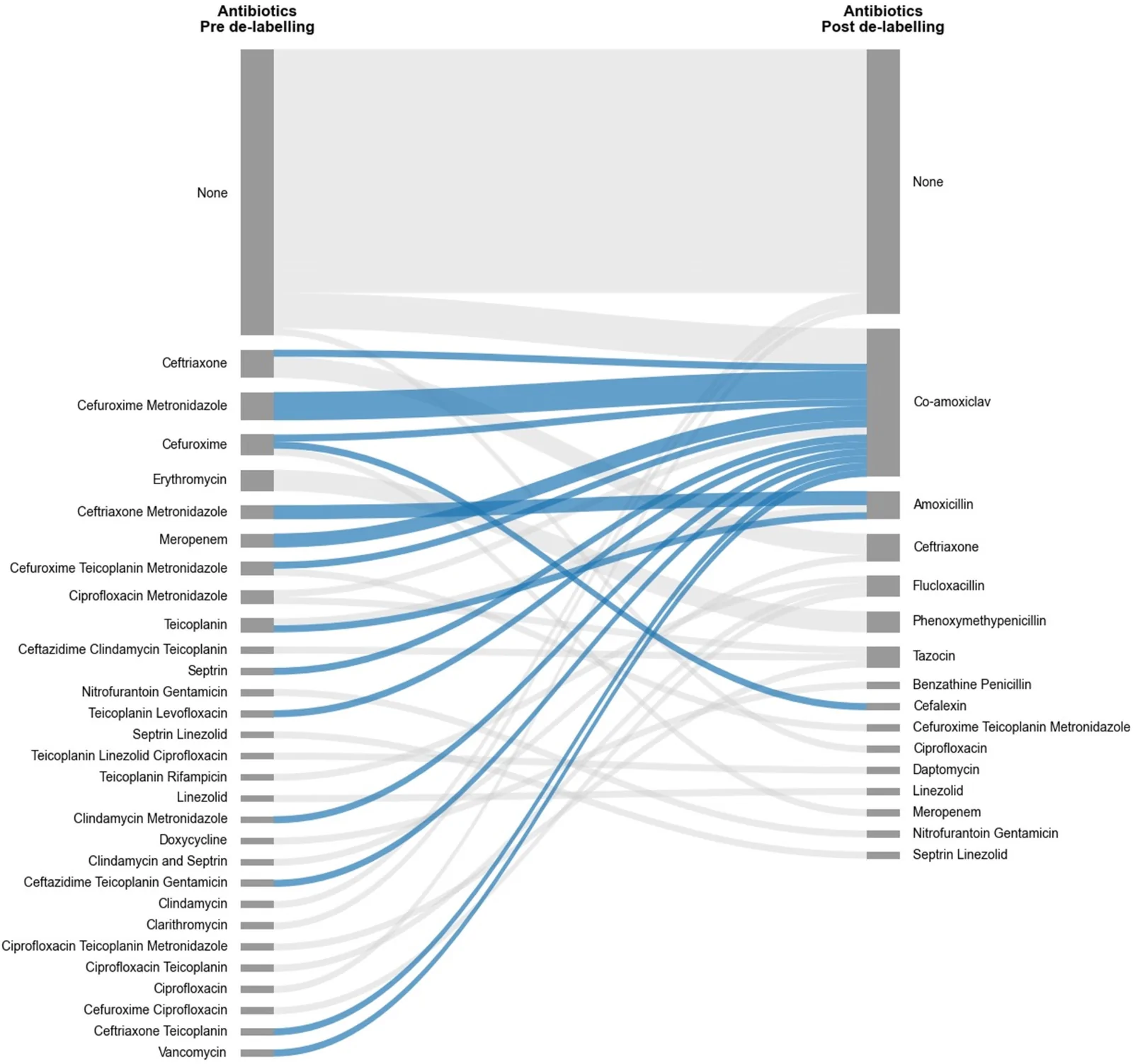
Antibiotics pre and post de-labelling. Blue lines denote IV to oral switch. Grey lines show not switched (e.g. IV to IV; oral to oral).

We assessed the impact of de-labelling on the 44 patients who were already on antibiotics at the time of review. In this cohort, de-labelling led to an immediate change in the antibiotic regimen in 84.1% (37/44) of cases (Table 3; Figure 4). Specifically, de-labelling facilitated an intravenous-to-oral switch in 43.2% (19/44) of patients, with 14 of these prescription changes occurring within 48 hours of the de-labelling review. Furthermore, the intervention resulted in a significant reduction in the mean number of antibiotics prescribed per patient, decreasing from 1.7 (standard deviation [SD] 0.71) to 1 (SD 0.50) (*P* = 0.00002). There was also a significant narrowing of antimicrobial breadth, with the mean ASC score decreasing from 9 (SD 4.6) to 6.2 (SD 3.4) (*P* = 0.00042).^27^ Of the remaining 41 patients who were not on antibiotics at the point of review, six patients were initiated on antibiotics as a direct result of the de-labelling review (*n* = 5, co-amoxiclav; *n* = 1 ciprofloxacin).

**Table 3.** Outcomes for patients who were on antibiotics at time of review

Outcomes post de-labelling	Patients on antibiotics at baseline (*n* = 44)
Any change in antibiotic regimen^a^, *n* (%)	37 (84.1)
IV to oral switch (IVOS)	
Within 48 hours *n* (%)	14 (31.8)
Within 3 months	5 (11.4%)
Total	19 (43.2%)
ASC Score	
Pre-de-labelling, mean ± SD	9 ± 4.6
Post-de-labelling, mean ± SD	6.2 ± 3.4
Mean difference	2.8
*P* value	0.00042
Number of antibiotics per patient	
Pre-de-labelling, mean ± SD	1.7 ± 0.7
Post-de-labelling, mean ± SD	1 ± 0.5
Mean difference	0.7
*P* value	0.00002

^a^Any change refers to IVOS/change in number or spectra of antibiotics prescribed. *P* values were calculated using the Wilcoxon signed-rank test.

### Comparison of BSACI and PEN-FAST criteria

Patient suitability for oral DPT was determined using the inclusion/exclusion criteria from the BSACI guidelines,^18^ although as a secondary datapoint the validated PEN-FAST penicillin allergy decision tool was considered.^18,19,29^ Overall, there was concordance in 65.2% (107/164) of cases; 85 were identified as low risk by both tools and proceeded to successful de-labelling by history alone/oral DPT, whereas 22 were identified as high risk by both tools (Table 4). Notably, nine of the 22 high-risk group completed full penicillin allergy by December 2025; four had their allergy confirmed and five were de-labelled. Of the cohort, 34.8% (57/164) were discordant, identified as high risk by BSACI criteria (warranting exclusion/referral for full penicillin allergy testing) but classified as low risk (score <3) by the PEN-FAST tool. Within this cohort, 21% (12/57) of patients completed onward full penicillin allergy testing by December 2025; nine were subsequently de-labelled and three patients had their allergy confirmed (Table 4).

**Table 4.** Comparison of the BSACI and PEN-FAST risk-stratification tools for triage to a direct oral drug provocation test

Risk-stratification category	Definition	Total (*n*)	Outcome from specialist referral		
			De-labelled	Allergy Confirmed	GP to refer/review in progress
Concordant low risk	BSACI criteria met AND PEN-FAST criteria met (score <3)	85	—	—	—
Concordant high risk	BSACI criteria NOT met AND PEN-FAST criteria NOT met (score ≥3)	22	5	4	13
Discordant	BSACI criteria NOT met AND PEN-FAST criteria met (score <3)	57	9	3	45
Total		164	14	7	58

### Expert review of cases

To understand the potential impact of a patient not being de-labelled, we randomly condensed 20 cases into short, anonymized, clinical vignettes. In each scenario, the penicillin allergy history was presented alongside guideline-based multiple-choice options for alternative management options. Vignettes were distributed via the British Infection Association mailing list, which is a national infection network.

Sixteen responses were received. These came primarily from consultants in microbiology (12/16, 75%), of whom two (12.5%) were retired. There was one response from an infection pharmacist, one from an infectious diseases consultant and two (12.5%) responses were from infection specialist trainees.

**Figure 5.**
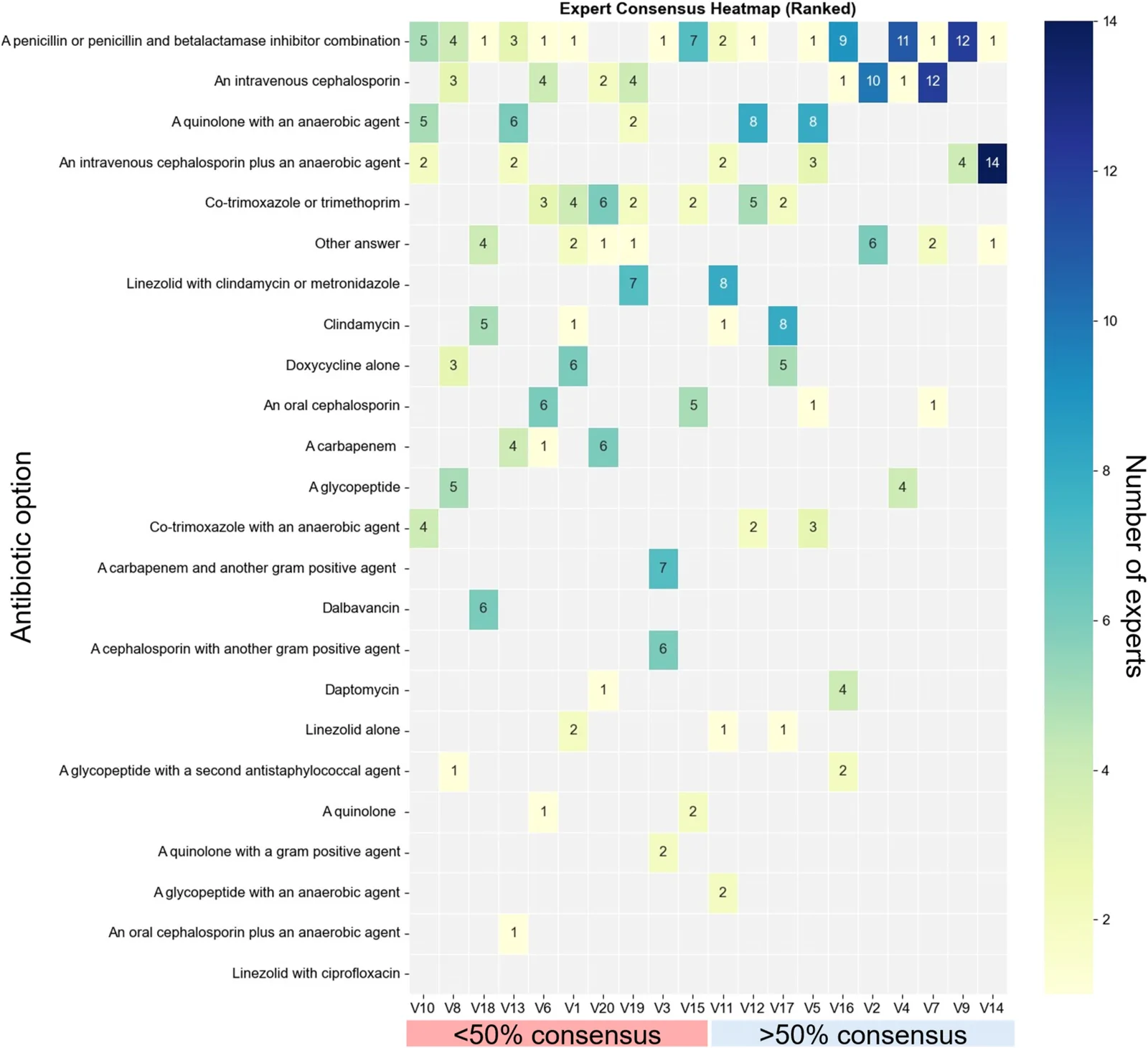
Heatmap shows expert consensus on theoretical antibiotic prescribing (had de-labelling not occurred) for each of 20 vignettes, ordered from lowest (left) to highest concordance (right).

In 10/20 vignettes, ≥50% consensus (i.e. ≥8 votes out of 16) was reached for a single treatment option (Figure 5), whereas no single option achieved a majority vote for the remaining 10 scenarios (Vignettes 10, 8, 18, 13, 6, 1, 20, 19, 3 and 15). Overall, expert opinions demonstrated minimal agreement (Fleiss’ kappa = 0.28) across all scenarios.

## Discussion

We demonstrate that our model of an embedded infection-led penicillin allergy de-labelling intervention presents a high-yield antimicrobial stewardship opportunity, resulting in conversion of IV to oral antibiotics in 43% of patients who were on antimicrobials before de-labelling, with concurrent reduction in the number and spectra of antibiotics. These interventions provide potential operational benefits including facilitation of early discharge and reduction in rates of antimicrobial associated infections such as *C. difficile*, which are more prevalent when second-line choices are used. Furthermore, our integrated infectious diseases review with de-labelling model potentially removes the requirement for separate outpatient allergy appointments, reducing healthcare costs. However, as a caveat, though our initial objective was to reduce burden on specialist allergy services by intercepting low-risk cases on the ward, 41.8% of our referrals did not meet low-risk criteria, prompting referral for full penicillin allergy testing, thus paradoxically increasing the absolute number of referrals allergy colleagues might receive. This highlights a crucial implementation lesson; non-specialist services must have robust, pre-agreed referral pathways with allergy colleagues to manage patients excluded from low-risk pathways. Notably, many of these patients would previously have received second-line agents without ever being referred to allergy services, so continuity of care within a low-risk pathway converts a proportion of them into new referrals. Onward referral was agreed with our allergy service at inception, and we are developing a business case for a joint infection-allergy post to meet this additional demand.

Our work further identifies that different risk-stratification metrics vary in their inclusivity of ‘low-risk’ cases. For example, criteria from BSACI guidelines led to the exclusion of 57 cases that would have been included by the PEN-FAST tool alone (score <3). Interestingly, 12/57 of this discordant group did go on to complete full penicillin allergy testing by December 2025; nine were confirmed not to have an allergy whereas three were confirmed to have either Type I or Type IV hypersensitivity reactions. These data therefore highlight the need for ongoing refinement of low-risk de-labelling triage tools, and potentially the benefit of consideration of more than one tool for decision making. For instance, PEN-FAST takes into account the number of years from the index reaction,^19,29^ a variable that has risk-stratification relevance given studies have shown diminishing skin test reactivity over time,^30^ while BSACI criteria cite helpful procedural exclusions (e.g. time from penicillin dose to rash <1 hour), which provide pragmatic indices for assessment of type 1 hypersensitivity. An unmet need for standardized guidelines is likely to be addressed by multi-site collaborative initiatives, for example through the International Network of Antibiotic Allergy Nations (iNAAN) Study (Australian New Zealand Clinical Trials Registry: ACTRN12623000484640). Locally, our pathway remains BSACI-based, with PEN-FAST recorded to support rather than determine decision making, pending standardized national guidance.

Our analysis further explored alternative management options had de-labelling not occurred. To this end, expert consensus on alternative prescribing was minimal. This might be partially driven by variations in individual clinicians’ risk tolerance regarding acceptance of reported penicillin allergy history (i.e. de-labelling based on history alone) and differing practices between hospital trusts. Nevertheless, these findings underscore that de-labelling promotes greater consistency in practice and increased use of first-line antibiotics, which are frequently penicillin-based. Indeed, our local hospital guidelines recommend penicillin-based regimens as first-line therapy for 56% of 101 distinct infection types.

While our study demonstrates the significant benefits of this infection-led approach, the following limitations should be considered. First, these implementation data derive from a single tertiary centre; although absolute service metrics will vary with local infrastructure and case-mix, the antimicrobial stewardship principles we describe, embedding de-labelling within existing infection touchpoints to drive intravenous-to-oral switch and spectrum narrowing, are not centre-specific, and our accompanying expert-opinion analysis drew on a national infection network. Prospective multi-centre evaluation would nonetheless be valuable to confirm generalizability. Second, while referral from non-infection specialties was possible, our service did not actively case-find patients not currently receiving antibiotics. These individuals might have benefited from proactive allergy de-labelling in anticipation of future antibiotic needs. This limitation was primarily due to resource constraints, which we aim to address in future service expansion. Similarly, patients excluded for clinical instability were re-reviewed only if they remained inpatients; a formalized route to recall these patients for later risk stratification is a gap we intend to address. Last, while the distribution of our case vignettes through a national infection email list aimed to minimize bias in expert selection, future work with larger datasets will seek to enhance the rigour of expert opinion through a Delphi method to achieve consensus. The vignette survey also drew 16 responses from a national network, and the resulting agreement estimates should be interpreted as indicative rather than definitive.

In sum, our study provides a compelling framework for an infection specialist-led penicillin allergy de-labelling service targeting patients with acute, complex or recurrent infections. This approach facilitates rapid antibiotic optimization, promotes antimicrobial stewardship, and has the potential to improve patient outcomes and reduce healthcare resource use.

## Supplementary Material

dlag189_Supplementary_Data

## Supplementary data

Supplementary Material available as Supplementary data at *JAC-AMR* Online.
