# Supplementary material for: Infection-embedded penicillin allergy de-labelling delivers immediate antimicrobial stewardship gains in patients with active infection
Source: JAC Antimicrob Resist. 2026 Sep 4;8(5):dlag189. doi: 10.1093/jacamr/dlag189 (PMC13542713; doi:10.1093/jacamr/dlag189)
Supplement: Supplementary file 1 [file dlag189_supplementary_data.docx]

**Supplementary Material:**

**Penicillin Allergy Clinical Vignettes**

**Survey Design:** This survey was designed to gather expert opinion from microbiologists, infectious diseases consultants, and pharmacists to determine the best ongoing treatment options for patients with complex or recurrent infections had they not been de-labelled by the penicillin allergy service. For each anonymised vignette, respondents were asked to choose the most appropriate option for ongoing management to facilitate discharge or outpatient care. The history of each index reaction to a penicillin is attached, and therefore, penicillins remain a treatment option in some cases (in the setting that the operator chooses to de-label on history alone).

**Scenario 1**

**Patient:** Female; age bracket 70-80

**Documented Allergies:**

- Penicillins (childhood reaction, no recollection of details)
- Metronidazole (rash and visual changes)

**Clinical Presentation:** 3 weeks post complex maxillary resection and reconstruction. After primary surgery, treated with clindamycin for seven days (initially IV then oral). Current issue: ?surgical site infection (no suspicion of osteomyelitis). Plan to discharge with oral antibiotics.

**Relevant History & Results:**

- **Results:** eGFR 80, normal potassium, no liver disease. MRSA negative. No swabs sent.

**Question:** What is the best option to facilitate discharge for treatment of an oral surgical site infection? **Options:**

- Co-trimoxazole 960mg BD PO
- Doxycycline 100mg BD PO
- Linezolid 600mg BD PO and ciprofloxacin 500mg BD PO
- Other

**Scenario 2**

**Patient:** Male; age bracket 20-30

**Documented Allergies:**

- Penicillins (rash with hives approx. 8 years ago)

**Clinical Presentation:** Diagnosed with late latent syphilis at a sexual health clinic. No neurosyphilis. Doxycycline discontinued due to severe diarrhoea.

**Relevant History & Results:**

- **Meds:** Carbamazepine, Levetiracetam.

**Question:** What is the best treatment option in this case? **Options:**

- Send stool for *C. difficile*; if negative, trial doxycycline 100mg PO BD again with loperamide.
- Ceftriaxone 2g IV OD in hospital for 14–21 days.
- Ceftriaxone 2g IV OD in outpatient setting (OPAT) for 14–21 days.
- Ceftriaxone 2g IM OD in outpatient setting for 14–21 days.
- De-sensitization to penicillin as inpatient followed by Benzathine penicillin 2.4 MU IM weekly for 3 weeks.
- Other

**Scenario 3**

**Patient:** Female; age bracket 40-50

**Documented Allergies:**

- Penicillins (delayed erythematous rash as a baby; no treatment required)

**Clinical Presentation:** Invasive Group A Strep infection, chest infection and mediastinitis requiring VATS debridement. Antibiotics during admission: Clindamycin/Meropenem/Linezolid (stopped due to eosinophilia)/Vancomycin/Ciprofloxacin. Current: Ceftazidime and Clindamycin.

**Relevant History & Results:**

- **Meds include:** Bisoprolol, Ramipril.
- **Microbiology:** Blood/tissue grew Group A Strep (sensitive to penicillins, erythromycin, clindamycin, doxycycline, linezolid). **Question:** What is the best treatment option currently pending further source control? **Options:**
- Continue ceftazidime 2g IV TDS and clindamycin 600mg IV QDS.
- Continue ceftazidime 2g IV TDS and change to linezolid 600mg IV or oral BD.
- Continue clindamycin 600mg IV QDS and change to ciprofloxacin 500mg IV TDS.
- Continue clindamycin 600mg IV QDS and trial meropenem 1g TDS again.
- Other

**Scenario 4**

**Patient:** Male; age bracket 30-40

**Documented Allergies:**

- Penicillins (unclear, recalls severe diarrhoea as inpatient on antibiotic)

**Clinical Presentation:** Long line infection. Ciprofloxacin/teicoplanin, rationalised to Teicoplanin 12mg/kg based on positive blood culture for Staphylococcus aureus; line removed. No endocarditis/bone involvement.

**Relevant History & Results:**

- **PMH:** CKD (baseline eGFR 10-15 mL/min)
- **Meds include:** Lansoprazole
- **Microbiology:** Blood culture: *S. aureus* (Resistant: Fusidic acid only).
- **Renal:** eGFR < 10 mL/min.

**Question:** What is the best treatment option? (Not well enough for discharge yet). **Options:**

- Continue on teicoplanin 12mg/kg OD IV.
- Switch to flucloxacillin equivalent of 2g QDS IV, adjusted for renal function.
- Change to vancomycin, dose adjusted for renal function/weight (aiming levels 15-20mg/L).
- Other

**Scenario 5**

**Patient:** Male; age bracket 60-70

**Documented Allergies:**

- Penicillins (delayed rash >40 years ago)

**Clinical Presentation:** Fevers, abdominal pain over several weeks. Admission blood cultures *E. coli* and *Clostridium species*; imaging revealed liver abscesses (multiple). Treated with cefuroxime/metronidazole with good response. Plan for discharge (4-6 weeks total).

**Relevant History & Results:**

- **PMH includes:** Crohn’s disease
- **Microbiology:** Blood culture: *E. coli* (Sensitive: Amoxicillin, Co-amoxiclav, Ceftriaxone, Ciprofloxacin, Trimethoprim, etc.).

**Question:** What is the best treatment option to facilitate discharge? **Options:**

- OPAT ceftriaxone 2g OD IV and oral metronidazole 400mg TDS.
- Oral ciprofloxacin 500mg BD and metronidazole 400mg TDS.
- Oral co-trimoxazole 960mg BD and metronidazole 400mg TDS.
- Oral cefalexin 500mg-1.5g BD-QDS and metronidazole 400mg TDS.
- Other

**Scenario 6**

**Patient:** Female; Age bracket 60-70 years

**Documented Allergies:**

- Penicillins (stomach pain, inability to walk)
- Intolerance to ciprofloxacin and trimethoprim (gastrointestinal disturbance)
- **Clinical Presentation:** Urosepsis, empirically treated with Ceftazidime/Teicoplanin/Gentamicin. Cultures grew *K. pneumoniae*. Nephrostomies exchanged. Patient refused ciprofloxacin.

**Relevant History & Results:**

- **PMH:** Bilateral nephrostomies secondary to malignancy. CKD.
- **Microbiology:** Blood/Urine: *K. pneumoniae* (Resistant: Amoxicillin, Piperacillin-Tazobactam. Sensitive: Co-amoxiclav, Ceftriaxone).
- **Question:** Best treatment to facilitate discharge (10-14 days total)? **Options:**
- Ciprofloxacin 500mg PO BD (manage side effects).
- Co-trimoxazole 960mg PO BD.
- Co-amoxiclav 625mg PO TDS.
- Ceftriaxone 2g IV OD via OPAT.
- Cefalexin 500mg-1.5g PO BD-QDS.
- Other

**Scenario 7**

**Patient:** Female: age bracket 60-70

**Documented Allergies:**

- Penicillins (rash and swelling as child)
- Meropenem (rash)

**Clinical Presentation:** Intraosseous abscess of femur. Underwent washout, treated with clindamycin then ciprofloxacin (switched to ceftriaxone 2g OD due to resistance). Recurrent accumulation required further washout. Discharged on OPAT Ceftriaxone (prolonged course due to chemotherapy and persistent infection on MRI).

**Relevant History & Results:**

- **PMH:** Haematological malignancy
- **Microbiology:** Femur tissue: *E. coli* (Resistant: Ciprofloxacin, Trimethoprim, Co-trimoxazole. Sensitive: Ceftriaxone, Ertapenem).

**Question:** Best treatment option to complete 3 months total? **Options:**

- Continue ceftriaxone 2g IV OD through OPAT.
- Cefalexin 500mg-1.5g PO TDS-QDS.
- Other

**Scenario 8**

**Patient:** Male; Age brackets 70-80 years

**Documented Allergies:**

- Penicillins (unknown, though doesn’t recall severe reaction)

**Clinical Presentation:** Developed septic arthritis of knee (red, hot, effusion). Washout performed; initial treatment with ceftriaxone. Wound swab grew *S. aureus*. Switched to teicoplanin. Plan for 4 weeks treatment.

**Relevant History & Results:**

- **PMH:** long term steroids
- **Microbiology:** Swab: *S. aureus* (Resistant: Erythromycin, Clindamycin. Sensitive: Flucloxacillin, Doxycycline, Tetracycline, Rifampicin, Ciprofloxacin). Knee aspirate: No growth

**Question:** Best treatment option for septic arthritis? **Options:**

- Doxycycline 200mg orally then 100-200mg daily.
- Ceftriaxone 2g IV OD through OPAT.
- Continue teicoplanin 12mg/kg OD through OPAT.
- Other

**Scenario 9**

**Patient:** Female: Age bracket 30-40

**Documented Allergies:**

- Penicillins (vomiting)

**Clinical Presentation:** Tubo-ovarian abscess (TOA). Empirically treated with Clindamycin/Meropenem/Metronidazole; poor response and re-presentation with new fever and non-drainable pelvis abscess on imaging. On treatment with Ciprofloxacin/Metronidazole.

**Relevant History & Results:**

- **Microbiology:** Fluid culture: Mixed anaerobes
- **Question:** Best treatment option to facilitate discharge (further 4 weeks)? **Options:**
- Ceftriaxone 2g OD IV + metronidazole 400mg TDS PO via OPAT.
- Co-amoxiclav 625mg TDS PO.
- Ciprofloxacin 500mg BD PO + metronidazole 400mg TDS PO.
- Other

**Scenario 10**

**Patient:** Male; age bracket 60-70

**Documented Allergies:**

- Penicillins (no recollection of events. Does not recall requirement for treatment or hospitalisation)

**Clinical Presentation:** Admitted with rigors and imaging revealed multiple liver abscesses, on a background of a stent in situ for cholangiocarcinoma that could not be removed. Treated with Teicoplanin/Ciprofloxacin/Metronidazole. Blood culture grew *E. coli*. Plan for 4 weeks of treatment.

**Relevant History & Results:**

- **Microbiology:** Blood: *E. coli* (Sensitive: Amoxicillin, Co-amoxiclav, Ciprofloxacin, Co-trimoxazole, Ceftriaxone).

**Question:** Best treatment option? **Options:**

- Ciprofloxacin 400mg BD PO and metronidazole 400mg TDS PO.
- Co-trimoxazole 960mg BD PO.
- Co-trimoxazole 960mg BD PO and metronidazole 400mg TDS PO.
- Ceftriaxone 2g OD IV and metronidazole 400mg TDS PO via OPAT.
- Co-amoxiclav 625mg TDS PO.
- Other

**Scenario 11**

**Patient:** Female; 60-70 age bracket.

**Documented Allergies:**

- Penicillins (non-severe rash)

**Clinical Presentation:** Osteomyelitis of left hemimandible post-reconstruction; debrided and metalwork removed. Treated with Cefuroxime/Metronidazole/Teicoplanin. Plan to rationalise for discharge (4 weeks total).

**Relevant History & Results:**

- **Microbiology:** Operative samples (mixed): *Streptococcus anginosus* (Res: Erythromycin. Sens: Clindamycin, Penicillin), *Actinomyces oris* (Sens: Penicillin, Clindamycin), *Haemophilus parainfluenza* (Sens: Co-trimoxazole, Co-amoxiclav).

**Question:** Best treatment option? **Options:**

- Teicoplanin 12mg/kg OD IV and metronidazole 400mg TDS PO via OPAT.
- Linezolid 600mg BD PO and metronidazole 400mg TDS PO (weekly bloods).
- Linezolid 600mg BD PO and clindamycin 300-450mg PO TDS-QDS (weekly bloods).
- Other

**Scenario 12**

**Patient:** Female; 50-60 age bracket

**Documented Allergies:**

- Penicillins (childhood rash)

**Clinical Presentation:** Recurrent cholangitis with biliary stent for cancer; ERCP declined. Treatment: Ceftazidime/Teicoplanin/Metronidazole.

**Relevant History & Results:**

- **Microbiology:** Blood culture: *Klebsiella sp.* (Resistant: Amoxicillin, Sensitive: Co-amoxiclav, Ceftriaxone, Ciprofloxacin, Co-trimoxazole).

**Question:** Best treatment option (short course)? **Options:**

- Ciprofloxacin 500mg BD PO +/- metronidazole 400mg TDS PO.
- Co-trimoxazole 960mg BD PO.
- Other

**Scenario 13**

**Patient:** Female; Age bracket 40-50

**Documented Allergies:**

- Penicillins (swelling)

**Clinical Presentation:** Readmitted several weeks after C-section with pyosalpinx. Poor response to Clindamycin/Metronidazole, escalated to Meropenem. Breastfeeding.

**Relevant History & Results:**

- **Microbiology:** *Group B Streptococcus* (Vaginal swab) and *E. coli* from urine (Res: Amoxicillin. Sens: Co-amoxiclav, Ceftriaxone, Ciprofloxacin).

**Question:** Best treatment option? **Options:**

- Ciprofloxacin 500mg BD PO and metronidazole 400mg TDS PO (advice re breastfeeding).
- Ceftriaxone 2g OD IV and metronidazole 400mg TDS PO via OPAT.
- Ertapenem 1g OD IV via OPAT.
- Co-amoxiclav 625mg TDS PO.
- Other

**Scenario 14**

**Patient:** Female; Age bracket 60-70

**Documented Allergies:**

- Penicillins (thrush)
- Macrolides (swelling)

**Clinical Presentation:** Started on Ciprofloxacin/Metronidazole for uncomplicated diverticulitis, developed new urticaria. Switched to Cefuroxime without reaction.

**Relevant History & Results:**

- **Microbiology:** No growth.

**Question:** Best treatment option (complete 7 days)? **Options:**

- Continue IV cefuroxime 1.5g TDS and metronidazole 500mg TDS PO as inpatient.
- Ceftriaxone 2g OD IV and metronidazole 500mg TDS PO via OPAT.
- Other

**Scenario 15**

**Patient:** Female; Age bracket 20-30

**Documented Allergies:**

- Penicillins (vomiting/unwell as child)

**Clinical Presentation:** Upper urinary tract infection, on background of continent urinary diversion.

**Relevant History & Results:**

- **Microbiology:** Urine: *E. coli* (Sensitive: All agents including Amoxicillin)

**Question:** Best treatment option (7-10 days)? **Options:**

- Ciprofloxacin 500mg BD PO.
- Co-trimoxazole 960mg BD PO.
- Trimethoprim 200mg BD PO.
- Cefalexin up to 1g QDS PO.
- Amoxicillin up to 1g TDS PO.
- Other

**Scenario 16**

**Patient:** Male; Age bracket 40-50 years

**Documented Allergies:**

- Penicillins (unclear, unable to report)

**Clinical Presentation:** Discitis and epidural collection, and Staphylococcus aureus bacteraemia; washout in theatre. Developed a rash on Teicoplanin/Rifampicin. Ongoing fevers and persistently high C-reactive protein.

**Relevant History & Results:**

- **Microbiology:** *S. aureus* (MSSA) (Sensitive: Flucloxacillin, Clindamycin, Linezolid. Resistant: Rifampicin).

**Question:** Best treatment option? (Cefazolin unavailable). **Options:**

- Continue teicoplanin 12mg/kg OD IV.
- Switch rifampicin to linezolid 600mg BD and continue teicoplanin.
- Switch to daptomycin 6-12mg/kg OD IV.
- Switch to flucloxacillin 2g 4-6 times a day IV.
- Other

**Scenario 17**

**Patient:** Male; Age bracket 70-80 years

**Documented Allergies:**

- Penicillins (rash)

**Clinical Presentation:** Diabetic foot infection and osteomyelitis. No collection/surgery needed. Plan: 6 weeks treatment.

**Relevant History & Results:**

- **Microbiology:** Previous swab: *Strep. dysgalactiae* (Sens: Clindamycin, Penicillin, Doxycycline) and *S. aureus* (Sens: Flucloxacillin, Doxycycline, Clindamycin).

**Question:** Best treatment option? **Options:**

- Clindamycin 300-450mg TDS-QDS PO.
- Co-trimoxazole 960mg BD PO.
- Linezolid 600mg BD PO with monitoring.
- Doxycycline 100mg BD PO.
- Other

**Scenario 18**

**Patient:** Female; Age bracket 50-60 years

**Documented Allergies:**

- Penicillins (chest pain, dizziness)

**Clinical Presentation:** Native valve endocarditis complicated by prosthetic material in situ. Treated with Teicoplanin. Plan: Lifelong suppression.

**Relevant History & Results:**

- **Microbiology:** Blood: *Strep. oralis/mitis* (Sens: Clindamycin, Penicillin, Teicoplanin, Ceftriaxone).

**Question:** Best treatment option for lifelong suppression? **Options:**

- Clindamycin at a low dose.
- Weekly or 2-weekly dalbavancin.
- Other

**Scenario 19**

**Patient:** Male; 50-60 years

**Documented Allergies:**

- Penicillins (rash)

**Clinical Presentation:** Cerebral abscess, subsequently drained. Treated with Ceftriaxone/Metronidazole (rationalised to Ceftriaxone). Plan: 6 weeks IV then oral step down (3 months total).

**Relevant History & Results:**

- **Microbiology:** Brain pus: *Actinomyces sp.* (Sens: Penicillin, Co-amoxiclav. Res: Metronidazole, Clindamycin). Molecular testing: *Fusobacterium nucleatum*.

**Question:** Best treatment option for oral consolidation? **Options:**

- Moxifloxacin 400mg OD PO +/- metronidazole 400mg TDS PO.
- Co-trimoxazole 960mg BD PO.
- Continue ceftriaxone 2g BD via OPAT.
- Linezolid 600mg PO BD +/- metronidazole 400mg TDS PO.
- Other

**Scenario 20**

**Patient:** Male; Age bracket 80-90 years

**Documented Allergies:**

- Penicillins (unknown)

**Clinical Presentation:** Spondylodiscitis and *S. aureus* bacteraemia. Treated with Teicoplanin, Linezolid, then Daptomycin, due to treatment failure.

**Relevant History & Results:**

- **Microbiology:** Blood: *Serratia marcescens* (AmpC) (Res: Amoxicillin, Co-amoxiclav, Ciprofloxacin. Sens: Ceftriaxone, Meropenem). *S. aureus* (Sens: Flucloxacillin, Daptomycin MIC 0.75).

**Question:** Best initial treatment option? **Options:**

- Meropenem 1g TDS IV as inpatient.
- Ceftriaxone 2g IV OD or BD.
- Daptomycin 6-12mg/kg OD IV.
- Teicoplanin 12mg/kg.
- Co-trimoxazole 960mg PO BD.
- Other
